# Quadratus Lumborum and Transversus Abdominis Plane Blocks and Their Impact on Acute and Chronic Pain in Patients after Cesarean Section: A Randomized Controlled Study

**DOI:** 10.3390/ijerph18073500

**Published:** 2021-03-28

**Authors:** Michał Borys, Aleksandra Zamaro, Beata Horeczy, Ewa Gęszka, Marek Janiak, Piotr Węgrzyn, Mirosław Czuczwar, Paweł Piwowarczyk

**Affiliations:** 1 Second Department of Anesthesia and Intensive Care, Medical University of Lublin, 20-059 Lublin, Poland; miroslaw.czuczwar@umlub.pl (M.C.); pawelpiwowarrczyk@umlub.pl (P.P.); 2 Department of Obstetrics and Perinatology, Faculty of Health Sciences, Medical University of Warsaw, 02-091 Warszawa, Poland; azamaro@wum.edu.pl (A.Z.); geszkae@gmail.com (E.G.); piotr.wegrzyn@wum.edu.pl (P.W.); 3 Anesthesiology and Intensive Care Department with the Center for Acute Poisoning, St. Jadwiga Provincial Clinical Hospital, 35-301 Rzeszów, Poland; beata.horeczy@yahoo.com; 4 First Department of Anesthesiology and Intensive Care, Medical University of Warsaw, 02-091 Warszawa, Poland; mjaniak8@gmail.com

**Keywords:** Neuropathic Pain Symptom Inventory, patient-controlled analgesia, quadratus lumborum block, transversus abdominis plane block, visual analog scale

## Abstract

Background: Severe postoperative pain is a significant problem after cesarean sections. Methods: This study was a randomized, controlled trial of 105 patients conducted in two hospitals. All patients were anesthetized spinally for elective cesarean section. Each participant was randomly allocated to one of three study groups: the quadratus lumborum block (QLB) group, the transversus abdominis plane block (TAPB) group, or the control (CON) group. The primary outcome of this study determined acute pain intensity on the visual analog scale (VAS). The secondary outcomes determined morphine consumption and chronic pain evaluation according to the Neuropathic Pain Symptom Inventory (NPSI) after hospital discharge. Results: At rest, the pain intensity was significantly higher in the CON group than in the QLB and TAPB groups at hours two and eight. Upon activity, the pain in the control subjects was more severe than in the QLB and TAPB groups in three and two of five measurements, respectively. Moreover, morphine consumption was significantly lower in the QLB (9 (5–10)) and TAPB (10 (6–14)) groups than in the CON (16 (11–19)) group. Persistent postoperative pain was significantly lower in the QLB group than in the CON group at months one and six following hospital discharge. Conclusions: Both the QLB and TAPB can improve pain management after cesarean delivery. Moreover, the QLB might reduce the severity of persistent postoperative pain months after cesarean section.

## 1. Introduction

Cesarean section is among the most common surgical procedures globally, and its use is on the rise in developed countries [1]. Most scheduled cesarean deliveries are performed under anesthesia using spinal or epidural techniques [2]. Although spinal anesthesia provides excellent postoperative analgesia after cesarean section, its effect endures for only a few hours after the surgery, after which patients can suffer from severe postoperative pain [3]. At that point, alternative pain management strategies should be implemented. One reason for this is an association between postoperative pain in patients after cesarean delivery and the risk of postpartum depression. Mothers with severe postoperative pain have a higher chance of postpartum depression, which can negatively affect a child’s development [4,5].

In recent years, novel ultrasound-guided regional analgesia techniques have been implemented in multimodal and rescue analgesia. The transversus abdominis plane block (TAPB) and the quadratus lumborum block (QLB) are new types of truncal plane blocks, and their roles in post-cesarean section pain management are still under investigation [6,7]. It has been argued that the TAPB reduces pain intensity and opioid consumption in the first 24 h after cesarean delivery when patients are anesthetized spinally; however, these effects were only observed if intrathecal opioid was not administered [8,9].

Several randomized controlled trials (RCTs) have investigated the role of the QLB in acute postoperative pain after cesarean section [10,11,12,13,14]. In all of these studies, the QLB reduced pain severity and opioid demand in comparison to the controlled group. However, different types of QLBs were used in these trials. In the RCTs by Blanco and Irwin, the QLB 2 or the posterior approach was utilized, while Mieszkowski and Krogh used the QLB 1 (anterolateral approach), and only in the study by Hansen et al. was the QLB 3 (transmuscular) performed. The only RCT in which the QLB was compared with the TAPB was conducted by Blanco and colleagues [15]. In that trial, the QLB 2 improved pain management quality, opioid consumption, and pain severity compared with the TAPB group. To our knowledge, none of the RCTs explored the impact of novel truncal blocks on persistent pain occurrence in patients after cesarean delivery.

Our study aimed to reevaluate the impact of the QLB and the TAPB on acute pain occurrence in patients after elective cesarean delivery. Chronic postoperative pain development and severity were also investigated among these individuals.

## 2. Materials and Methods

### 2.1. Study Design

This prospective, randomized, double-blind trial was conducted in two obstetric departments of a secondary and a tertiary hospital. The study protocol was approved by the Institutional Review Board of the Medical University of Lublin (KE-0254/127/2017) and was prospectively registered at ClinicalTrials.gov (NCT03244540, 8 August 2017). Informed, written consent was obtained from every patient, and the study methods met the tenets of the Declaration of Helsinki for medical research involving human subjects. The first patient was recruited on 4 September 2017.

Only singleton pregnancy females, older than 18 years, scheduled for a cesarean section under spinal anesthesia could participate in the study. The exclusion criteria included a lack of informed consent, allergy to drugs used during the study, depression and epilepsy that required antidepressants or anticonvulsants, known coagulopathy as a contraindication for spinal anesthesia, addiction to alcohol or recreational drugs, and gestational age below 36 weeks.

### 2.2. Procedure

Each patient received single-shot spinal anesthesia with 0.5% hyperbaric bupivacaine (Marcaine Spinal 0.5% Heavy, Aspen Pharma Trading Limited, Dublin, Ireland) without an additional opioid. The bupivacaine solution was administered according to the patient’s height: from 1.8 mL for 150 cm individuals to 2.6 mL for 180 cm patients. At the end of the cesarean section, patients were randomly allocated to one of three groups (1:1:1 randomization): the QLB, TAPB, or control group (CON). The team member who anesthetized the patient opened an opaque envelope in which an allocation group was indicated. The envelope was sealed, and the patient allocation was prepared according to the randomization by a team member not directly involved in the process of anesthesia or further evaluation of the participants.

**Figure 1:**
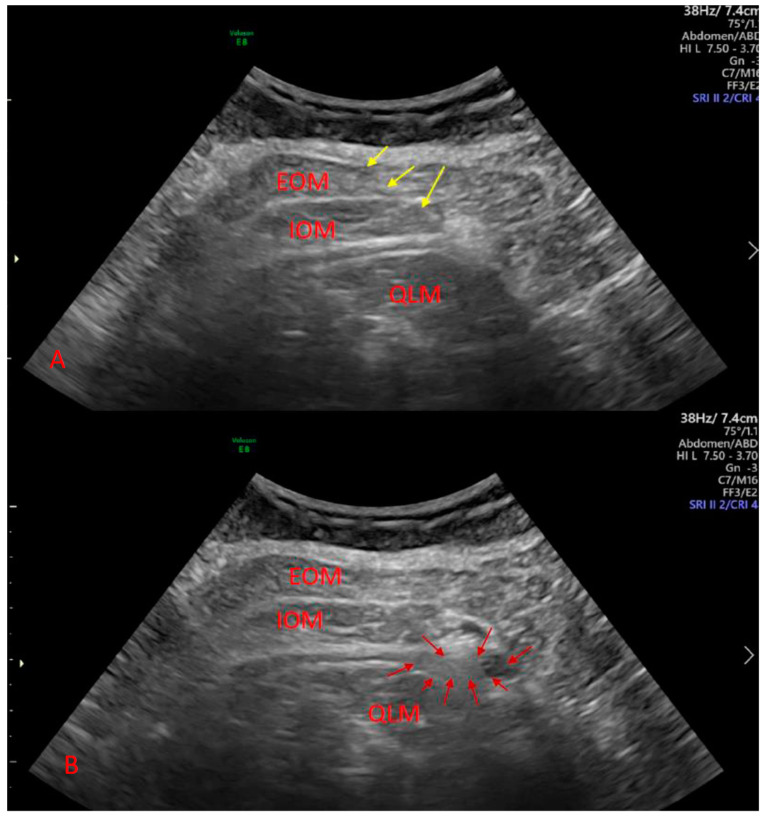
Ultrasound-guided quadratus lumborum block.

The plane blocks were performed in the operating room before the transfer of patients to the ward. A curvilinear ultrasound probe was used to identify the truncal muscle and perform the QLB. In this group, a material wedge was placed under the patient’s back at the site of injection. The type 2 QLB (posterolateral) was performed in each case (Figure 1). To identify the abdominal muscle in the TAPB group, a linear or convex probe was used. The lateral TAPB was performed in this case. An ultrasound-dedicated needle (Stimuplex Ultra 360, B. Braun, Melsungen, Germany) was utilized to perform the procedure. Each patient received a solution of 0.375% ropivacaine (Ropimol, Molteni, Italy), 0.2 mL per kg (up to 20 mL), on each side. In the CON group, the anesthesiologist pretended that the block was performed. The ultrasound machine was in the operating room and the probe was placed on the abdominal wall. The patients did not see the operating field. All procedures including truncal and spinal blocks were performed by three experienced anesthesiologists.

Sonographic image of quadratus lumborum block before (A) and after (B) local anesthetic deposition. Yellow arrows denote needle shaft, and red arrows denote local anesthetic. EOM denotes external oblique muscle, IOM denotes internal oblique muscle, and QLM denotes quadratus lumborum muscle.

### 2.3. Pain Management

In the postoperative care unit, pain treatment with a patient-controlled analgesia pump was initiated. A solution of morphine (Morphini sulfas WZF, Warszawa, Poland), 1 mg/mL, 1 mL per bolus with a lockout time of five minutes was administered. In the case of severe pain, more than 40 on the visual analog scale (VAS), an additional bolus of morphine (5 mg) could be administered at the midwife’s discretion. Moreover, each patient received intravenous paracetamol (Paracetamol Kabi, Fresenius Kabi Polska, Warszawa, Poland) every six hours.

### 2.4. Outcomes

The primary outcome of this study determined acute pain intensity measured at rest and upon activity on the VAS (0 to 10 cm) at 2, 4, 8, 12, and 24 h after the cesarean section. Pain upon activity was measured during the abdominal muscle flexion, a trial of sitting up or raising the legs if the motor block vanished. The pain intensity was measured by an anesthesiologist who was not aware of the patient allocation (blinding).

The other outcomes included morphine consumption during the first 24 h and chronic pain evaluation via the Neuropathic Pain Symptom Inventory (NPSI) developed by Bouhassira et al. [16]. This inventory consists of a list of descriptors reflecting spontaneous pain, evoked pain, and dysesthesia/paresthesia. Each of the items was quantified on a (0–10) numerical rating scale (NRS). The pain severity was presented as the sum of 10 descriptors from 0 to 100. Moreover, a number of patients with any signs of chronic pain was shown. Persistent pain severity was also presented for these individuals who had any signs of pain. Patients without pain were omitted from this analysis (adjusted analysis). The study participants were interviewed over the phone to assess the prevalence and characteristics of chronic pain. The NPSI was used in our previous study concerning persistent postoperative pain in patients after cesarean section [17]. The full questionnaire is presented as an additional file (Table S1).

### 2.5. Statistical Analysis and Sample Size Calculation

The normality of the distribution was investigated for each continuous variable with the Shapiro–Wilk test. Normally distributed parameters were analyzed using an analysis of variance (ANOVA) method. These variables are presented as means with 95% confidence intervals. The Kruskal–Wallis test by ranks was used to compute parameters with non-normal distributions. A pairwise comparison was performed with the Mann–Whitney U test, and the Bonferroni correction was applied if the results of the Kruskal–Wallis test showed statistical significance. These data are presented as medians and interquartile ranges (IQR). Qualitative parameters were compared with the Fisher’s exact test. Logistic regression was used to reveal the parameters that affect chronic pain presentation, and the odds ratio (OR) was used to describe the predictors that were included in the model. The receiver operating characteristic (ROC) curve was calculated for the best model. All measurements were performed using Statistica 13.1 software (StatSoft, Tulsa, OK, USA). The randomization was also generated with Statistica software’s random number generator by a team member who was not directly involved in the process of recruiting, treating, and assessing patients.

A preliminary study was performed to assess the sample size. The primary outcome of the study was the acute pain measurement on the VAS. The sample size analysis was calculated for the acute pain at hour 2. Thirty patients, 10 per group, were included with the mean VAS results 1, 1.5, and 2 in QLB, TAPB, and QLB, respectively. The calculated sample size for different means was 27 individuals for each group, with a root mean square standardized effect of 0.5, power 0.9, and alpha 0.05. We decided to randomize 105 participants into groups with 35 individuals in each group.

## 3. Results

**Figure 2:**
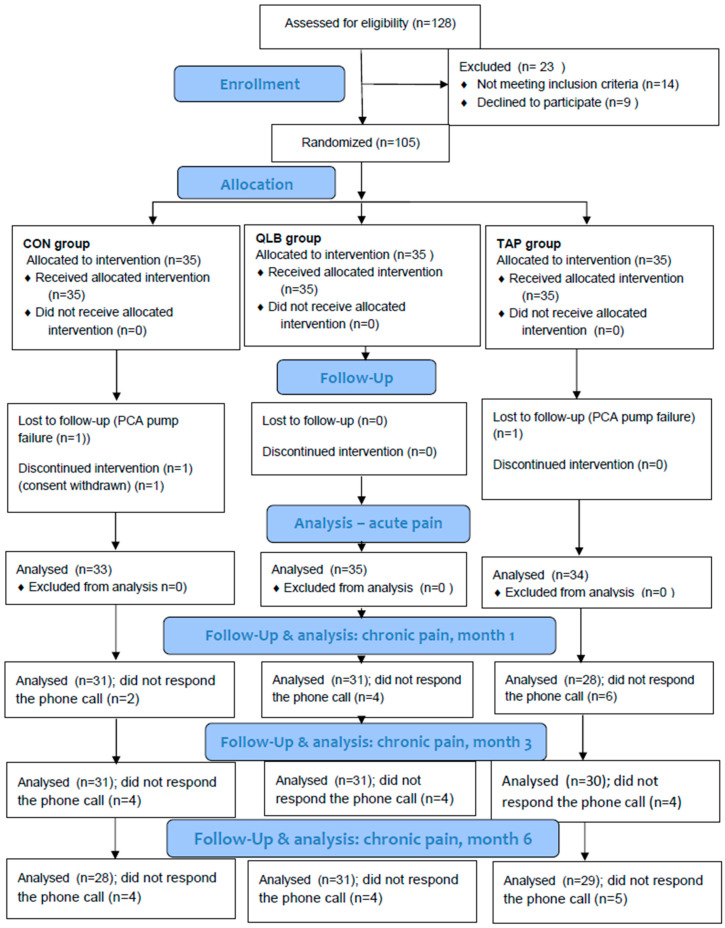
Study flowchart.

The study was conducted from September 2017 to August 2019. Of the 128 patients assessed for eligibility, 105 were randomized and allocated to the intervention. A detailed description of the study participants is presented in the flowchart (Figure 2).

CON denotes the control group, PCA denotes patient-controlled analgesia, QLB denotes the quadratus lumborum block group, and TAPB denotes the transversus abdominis plane block group.

Patient demographic data and indications for cesarean delivery are shown in Table 1. A statistically significant difference was found in the psychological indications for cesarean delivery.

### 3.1. Acute Pain

Of the five pain measurements at rest, a significant difference was found at a single time point (Table 2). The pain intensity was significantly lower in the QLB group than in the CON group at hour two and lower in the QLB group than in the TAPB group at hour eight.

At three time points, a statistically significant difference was found upon activity (Table 3). Lower pain intensity was observed in the QLB and TAPB groups than in the CON group at hours 2 and 24. Moreover, lower pain severity was detected in the QLB group than in the CON group at hour 12.

**Figure 3:**
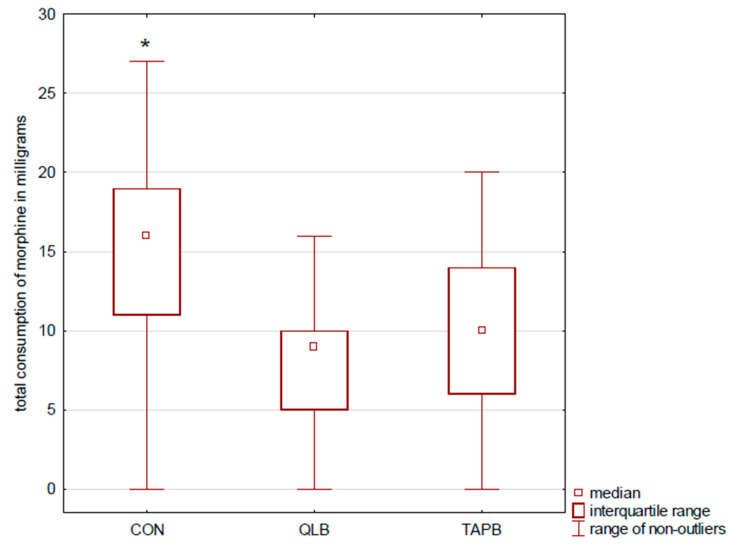
Morphine consumption. The figure presents morphine consumption via PCA pumps during the first postoperative occurrence of pain. Results are presented as medians and interquartile ranges. * indicates statistical significance, CON > QLB, and TAPB. CON denotes the control group, PCA denotes patient-controlled analgesia, QLB denotes the quadratus lumborum block group, and TAPB denotes the transversus abdominis plane block group.

Patients in the QLB and TAPB (9 mg IQR (5–10) and 10 mg (6–14)) groups used less morphine via PCA than participants in the CON group (16 mg (11–19); *p* < 0.001) (Figure 3). Moreover, controls used statistically more rescue morphine boluses (0 (0–5); *p* < 0.001) than patients after the truncal blocks. No difference was found between the QLB and TAPB groups in postoperative pain severity and morphine consumption.

### 3.2. Chronic Pain

The pain severity measured via the NPSI was significantly lower at months one and six in the QLB group than in the CON group (Table 4). However, no difference was noted in persistent pain severity between the QLB and TAPB groups. Moreover, we did not find the difference between the TAPB and CON at any evaluation of chronic pain intensity. 

No difference was detected between the QLB and TAPB groups. Moreover, no difference was found between the study groups in the number of patients who had any sign of chronic pain (Table 5).

### 3.3. Chronic Pain Predictors

The factors associated with chronic pain occurrence after cesarean delivery included persistent pain severity at month three, previous cesarean section, and fetal malpresentation. Severe pain at month three increased the risk of chronic pain prevalence at month six (OR 1.28 (1.1, 1.49), *p* = 0.001). Conversely, subsequent cesarean section and fetal malpresentation decreased the risk of chronic pain prevalence at month six. The calculated OR was 0.225 (0.083, 0.613), *p* < 0.01 for the previous cesarean delivery and 0.036 (0.002, 0.79), *p* = 0.036 for fetal malpresentation. The area under the ROC curve for this model was 0.892.

## 4. Discussion

The results presented in the current study showed an alleviation of pain severity in patients after plane blocks in comparison with the CON group. Pain intensity upon activity was statistically decreased at two of the five time points in the TAPB group and three of the five measurements in the QLB group. However, at rest, the only significant difference was observed between patients after plane blocks and the CON group during a single pain evaluation. Moreover, patients in the QLB and TAPB groups used less morphine via PCA during the first postoperative day than participants in the CON group. In the current study, no difference was found between the TAPB and QLB groups in pain intensity or morphine consumption. The long-term evaluation showed a significant difference in chronic pain severity between the QLB and CON groups at months one and 6six However, according to the International Association for the Study of Pain, chronic pain can be recognized at least three months after surgery [18]. Moreover, no difference was noticed regarding chronic pain severity between the QLB and the TAPB groups or the CON and the TAPB groups.

To our knowledge, the only study that directly compared QLB with TAPB after cesarean section was conducted by Blanco et al. [15]. In results similar to ours, the authors of that trial did not find any difference between the two truncal blocks in pain severity measured via VAS. However, in that study, morphine consumption was significantly lower in the QLB group than in the TAPB group after 24 h and was 6 mg (4.75–16) vs. 16.5 mg (8–33.25). Interestingly, the morphine consumption reported by Blanco et al. in their TAPB group was similar to that of the CON group in the present study (16 mg (11–19)).

The results of a recent metanalysis by El-Boghdadly et al. showed that both the QLB and the TAPB were superior to control in the absence of intrathecal morphine [19]. However, when intrathecal morphine was administered, no difference was found between the truncal blocks and control. In the current study, we decided not to add an opioid to the hyperbaric bupivacaine during the spinal blocks because of differences between both hospitals where the study was performed according to the anesthetic regime. Despite that, in the current study, the total consumption of morphine during the first 24 h in the CON group (median 16, mean 14.4 mg) was similar to consumption found in other trials. In the study conducted by Blanco et al., the mean consumption of morphine in the CON group was 19 mg while in the trials conducted by Irwin et al. and Mieszkowski et al., it was 14 mg and 17 mg, respectively [10,11,12].

Several trials have evaluated the efficacy of QLB analgesia after cesarean section, including QLB 1 (anterolateral), QLB 2 (posterior), and QLB 3 comparing the block with the control (sham or no block) [10,11,12,13,14,15]. There were some methodological differences between these studies, including a different injection site (different QLBs), ketobemidone used as an opioid in the study by Krogh et al., the lack of a sham block in the study by Mieszkowski et al., a different type of local anesthetic used for the block (bupivacaine, levobupivacaine, ropivacaine), and different opioids (sufentanil, fentanyl, morphine) added to hyperbaric bupivacaine during the spinal anesthesia; however, all these reports presented decreased opioid consumption and moderate pain relief in patients after the plane block compared with the CON group.

In our previous study, we assessed the prevalence and severity of chronic pain in patients who received QLB or TAPB after cesarean delivery [17]. This observational trial did not present any difference in chronic pain intensity between the plane block and CON groups; however, despite the large number of recruited participants, that trial had many limitations, including suboptimal management of postoperative pain (e.g., a lack of opioids administered via PCA pumps). Moreover, the absence of randomization could have caused selection bias. In the current study, most of these issues were eliminated.

Only a few studies have presented the alleviation of persistent postoperative pain due to regional anesthesia techniques [20,21,22]. In these studies, the paravertebral block or the epidural anesthesia reduced chronic pain intensity in patients after a thoracotomy or breast surgery. In each of these studies, the regional block was performed preemptively. To our knowledge, the only RCT in which the regional anesthesia technique reduced the risk of persistent postoperative pain was conducted by Shahin and Osman [23]. The authors of this study injected 10 mL of 2% lidocaine or 10 mL of 0.9% NaCl intraperitoneally at the end of the cesarean section. Eight months after the surgery, fewer patients in the study group had incidents of persistent postoperative pain, 20.8% versus 10.8%, and lower pain intensity measured via NRS was noticed after intraperitoneal lidocaine injection than in the CON group. In the present study, pain severity was significantly lower at month six in the QLB group than in the CON group. Although statistical significance was reached regarding chronic pain intensity, even after the adjustment of these results, the median values of pain severity at month six were relatively low (Table 4). However, we did not find lower numbers of patients with chronic pain in any group. In this study, we decided to use the NPSI to assess persistent pain severity [16]. This inventory is multi-dimensional and covers many aspects of chronic pain. We used the NPSI in our previous studies for persistent pain measurement [17,22]. Moreover, NPSI were used by other authors to measure persistent postoperative pain [24,25,26]. As presented by Yazici et al., the surgical technique may affect the prevalence of neuropathic pain in patients following the cesarean section [27]. Thus, the inventory focused on neuropathic pain measurement, such as the NPSI, might be useful in this population of patients.

The result of the logistic regression showed that three variables were associated with chronic pain prevalence in our trial. Only pain severity at month three increased the risk of chronic pain occurrence at month six. Both fetal malpresentation and subsequent cesarean section were associated with a lower prevalence of persistent pain. Our results are consistent with the findings presented by Niklasson et al. [28]. The authors of that paper found that the first cesarean delivery was a risk factor for increased chronic pain at month six. In contrast to our results, Wang et al. showed that having a previous cesarean section was associated with a higher risk of persistent pain [29]. This discrepancy may be the result of differences in cultural backgrounds.

Although our trial was preceded by a pilot study and power calculation was performed, still, a sample size could be not sufficient. In the study by McKeen et al. comparing the TAPB with controls in patients after the cesarean section, the mean NRS at hour 2 was 2.0 in the studied and 3.5 in the placebo group with a standard deviation of 2.3 [30]. If the sample size in our study had been calculated according to data from the RCT by McKeen and colleagues, we would have included approximately a hundred participants per group.

Our study had some limitations. Because QLB and TAPB were performed after the administration of spinal anesthesia, we did not test the block area with the pinprick technique. The study was conducted in two hospitals; therefore, truncal blocks were done by three anesthesiologists. We did not add any opioid spinally which could have affected pain severity after the procedure. Although statistical significance was detected for many parameters and the recruitment period was preceded by the power analysis, the number of analyzed patients was still limited. Despite the power calculation, our study could be underpowered regarding the primary outcome. The results of this study showed difference between the CON and the QLB groups in persistent pain severity; however, the median values of pain severity were relatively low.

## 5. Conclusions

Our study showed that both truncal blocks reduced pain severity and total morphine consumption compared with the controls in patients after cesarean delivery. However, no difference in acute postoperative pain was observed between the QLB and TAPB groups. Moreover, patients in the QLB group had lower pain intensity than those in the CON group six months after surgery. It appears that the QLB might alleviate chronic postoperative pain severity in patients after cesarean section.

## Figures and Tables

**Table 1:** Patient demographics and indications for cesarean section.

Groups (N)	QLB (N = 35)	CON (N = 33)	TAPB (N = 34)	*p*-Value
Age (years)	30.7 ± 4.0	32.5 ± 5.7	31.4 ± 5.9	0.37
Height (cm)	168.3 ± 7.1	165.7 ± 6.4	169.4 ± 7.6	0.10
Weight (kg)	79.6 ± 13.8	80.2 ± 9.5	76.4 ± 11.0	0.36
Subsequent cesarean section	20 (57.1)	14 (42.4)	10 (29.4)	0.35
Failure to progress	6 (17.1)	3 (9.1)	6 (17.6)	0.65
Nonreassuring fetal heart rate tracing	1 (2.9)	4 (12.1)	3 (8.8)	0.53
Fetal macrosomia	2 (5.7)	6 (18.2)	1 (2.9)	0.14
Psychological	2 (5.7)	0 (0)	8 (23.5)	<0.01
Fetal malpresentation	2 (5.7)	5 (15.2)	2 (5.9)	0.50
Other	2 (5.7)	1 (3.0)	4 (11.8)	0.50

Age, height, and weight are presented as mean and standard deviations. The probability for these variables was calculated using one-way ANOVA. Indications for cesarean section are presented as N and (%). The probability was calculated with the Freeman–Halton extension of Fisher’s exact test. N denotes sample size, CON denotes the control group, QLB denotes the quadratus lumborum block group, and TAPB denotes the transversus abdominis plane block group.

**Table 2:** Pain at rest.

Group (N)	QLB (N = 35)	CON (N = 33)	TAPB (N = 34)	*p*-Value
VAS 2	1 (0–2) ^a^	3 (2–4)	2 (0–3)	<0.001
VAS 4	2 (0–4)	3 (3–4)	3 (1–4)	0.2
VAS 8	2 (0–3) ^b^	3 (2–4)	3 (2–5)	0.027
VAS 12	2 (1–4)	4 (2–5)	3 (2–4.6)	0.18
VAS 24	2 (0–4)	2.5 (1–5)	3 (1–4)	0.15

The table presents the subsequent results of visual analog scale intensity in mm after the cesarean section. Data are shown as medians (interquartile ranges). The probability was calculated with the Kruskal–Wallis test by ranks. If this test showed a significant result, a pairwise comparison was made with the Mann–Whitney U test. A significant calculated probability was set at 0.017 after Bonferroni correction; ^a^: QLB < CON; ^b^: QLB < TAPB. CON denotes the control group, QLB denotes the quadratus lumborum block group, and TAPB denotes the transversus abdominis plane block group.

**Table 3:** Pain upon activity.

Group (N)	QLB (N = 35)	CON (N = 33)	TAPB (N = 34)	*p*-Value
VAS 2	2 (0–3) ^a^	3 (3–5)	2 (1–3) ^b^	<0.001
VAS 4	4 (3–5)	4 (4–5)	3 (2–5)	0.048
VAS 8	4 (2–5)	4 (4–5)	4 (3–5)	0.17
VAS 12	4 (2–5) ^a^	5 (4–6)	4 (3–5.4)	0.013
VAS 24	4 (2–5.25) ^a^	5 (4–6)	4 (3.15–5.25) ^b^	<0.01

The table presents the subsequent results of visual analog scale intensity in mm after the cesarean section. Data are shown as medians (interquartile ranges). The probability was calculated with the Kruskal–Wallis test by ranks. If this test showed a significant result, a pairwise comparison was made with the Mann–Whitney U test. A significant calculated probability was set at 0.017 after Bonferroni correction; ^a^: QLB < CON; ^b^: TAPB < CON. CON denotes the control group, QLB denotes the quadratus lumborum block group, and TAPB denotes the transversus abdominis plane block group.

**Table 4:** Persistent pain severity.

Time of Evaluation	QLB	CON	TAPB	*p*-Value
Month 1	0 (0–4) ^a^	12 (0–22)	3.5 (0–9.5)	<0.01
Month 3	0 (0–6)	2 (0–23)	3.5 (0–7)	0.21
Month 6	0 (0–6) ^a^	4.25 (0–13)	0 (0–8)	0.039

The severity of chronic pain detected with NPSI (0–100) is reported as medians (interquartile ranges). The probability was calculated with the Kruskal–Wallis test by ranks. If this test showed a significant result, a pairwise comparison was made with the Mann–Whitney U test. A significant calculated probability was set at 0.017 after Bonferroni correction; a: QLB < CON. CON denotes the control group, QLB denotes the quadratus lumborum block group, and TAPB denotes the transversus abdominis plane block group.

**Table 5:** Number of patients with persistent pain.

Time of Evaluation	QLB	CON	TAPB	*p*-Value
Month 1	14/31	23/31	17/28	0.034
Month 3	15/31	19/31	18/30	0.56
Month 6	10/31	16/28	13/29	0.158

The table presents the number of patients who perceived any signs of chronic postsurgical pain at one, three, and six months after cesarean section. The probability was calculated with the Freeman–Halton extension of Fisher’s exact test. CON denotes the control group, QLB denotes the quadratus lumborum block group, and TAPB denotes the transversus abdominis plane block group.

## Supplementary Materials

The following are available online at https://www.mdpi.com/article/10.3390/ijerph18073500/s1, Table S1: The Neuropathic Pain Symptom Inventory (NPSI).
