# Supplementary material for: Quadratus Lumborum and Transversus Abdominis Plane Blocks and Their Impact on Acute and Chronic Pain in Patients after Cesarean Section: A Randomized Controlled Study
Source: Int J Environ Res Public Health. 2021 Mar 28;18(7):3500. doi: 10.3390/ijerph18073500 (PMC8037180; doi:10.3390/ijerph18073500)
Supplement: Supplementary file 1 [file ijerph-18-03500-s001.pdf]

Neuropathic pain syndrome inventory, according to Bouhassira D, Attal N, Fermanian J, et al. Development and validation of the Neuropathic Pain Symptom Inventory. Pain. 2004;108(3):248-257.

We wish to know if you feel spontaneous pain, that is pain without any stimulation. For each of the following questions, please select the number that best describes your average spontaneous pain severity during the past 24 h. Select the number 0 if you have not felt such pain (circle one number only).

1. Does your pain feel like burning?

    No burning 0 1 2 3 4 5 6 7 8 9 10 Worst burning imaginable

2. Does your pain feel like squeezing?

    No squeezing 0 1 2 3 4 5 6 7 8 9 10 Worst squeezing imaginable

3. Does your pain feel like pressure?

    No pressure 0 1 2 3 4 5 6 7 8 9 10 Worst pressure imaginable

4. During the past 24 h, your spontaneous pain has been present:

   Select the response that best describes your case

   a. Permanently
   b. Between 8 and 12 h
   c. Between 4 and 7 h
   d. Between 1 and 3 h
   e. Less than 1 h

We wish to know if you have brief attacks of pain. For each of the following questions, please select the number that best describes the average severity of your painful attacks during the past 24 h. Select the number 0 if you have not felt such pain (circle one number only).

5. Does your pain feel like electric shocks?

    No electric shocks 0 1 2 3 4 5 6 7 8 9 10 Worst electric shocks imaginable

6. Does your pain feel like stabbing?

    No stabbing 0 1 2 3 4 5 6 7 8 9 10 Worst stabbing imaginable

7. During the past 24 h, how many of these pain attacks have you had?

   Select the response that best describes your case

   a. More than 20
   b. Between 11 and 20
   c. Between 6 and 10
   d. Between 1 and 5

e. No pain attack

We wish to know if you feel pain provoked or increasedby brushing, pressure, contact with cold or warmth on the painful area. For each of the following questions, please select the number that best describes the average severity of your provoked pain during the past 24 h. Select the number 0 if you have not felt such pain (circle one number only).

8. Is your pain provoked or increased by brushing on the painful area?

No pain 0 1 2 3 4 5 6 7 8 9 10 Worst pain imaginable

9. Is your pain provoked or increased by pressure on the painful area?

No pain 0 1 2 3 4 5 6 7 8 9 10 Worst pain imaginable

10. Is your pain provoked or increased by contact with something cold on the painful area?

No pain 0 1 2 3 4 5 6 7 8 9 10 Worst pain imaginable

We wish to know if you feel abnormal sensations in the painful area. For each of the following questions, please select the number that best describes the average severity of your abnormal sensations during the past 24 h. Select the number 0 if your have not felt such sensation (circle one number only).

11. Do you feel pins and needles?

No pins and needles 0 1 2 3 4 5 6 7 8 9 10 Worst pins and needles imaginable

12. Do you feel tingling?

No tingling 0 1 2 3 4 5 6 7 8 9 10 Worst tingling imaginable
